# Assessing the presence of *Wuchereria bancrofti* in vector and human populations from urban communities in Conakry, Guinea

**DOI:** 10.1186/s13071-015-1077-x

**Published:** 2015-09-26

**Authors:** Bernard L. Kouassi, Dziedzom K. de Souza, Andre Goepogui, Charles A. Narh, Sandra A. King, Baldé S. Mamadou, Lamia Diakité, Samuel K. Dadzie, Daniel A. Boakye, Jürg Utzinger, Moses J. Bockarie, Benjamin G. Koudou

**Affiliations:** Centre Suisse de Recherches Scientifiques en Côte d’Ivoire, Abidjan, Côte d’Ivoire; Unité de Formation et de Recherche Science de la Nature, Université Nangui Abrogoua, Abidjan, Côte d’Ivoire; Department of Epidemiology and Public Health, Swiss Tropical and Public Health Institute, Basel, Switzerland; University of Basel, Basel, Switzerland; Parasitology Department, Noguchi Memorial Institute for Medical Research, University of Ghana, Legon, Accra, Ghana; Programme National de Lutte contre l’Onchocercose, le Trachome et les autres Maladies Tropicales Négligées, Ministère de la Santé Publique, Conakry, Guinea; Centre for Neglected Tropical Diseases, Liverpool School of Tropical Medicine, Liverpool, UK

**Keywords:** Guinea, Lymphatic filariasis, Mass drug administration, Transmission, *Wuchereria bancrofti*

## Abstract

**Background:**

The Global Programme to Eliminate Lymphatic Filariasis was launched in 2000 with the goal of interrupting transmission of lymphatic filariasis (LF) through multiple rounds of mass drug administration (MDA). In Guinea, there is evidence of ongoing LF transmission, but little is known about the most densely populated parts of the country, including the capital Conakry. In order to guide the LF control and elimination efforts, serological and entomological surveys were carried out to determine whether or not LF transmission occurs in Conakry.

**Methods:**

The prevalence of circulating filarial antigen (CFA) of *Wuchereria bancrofti* was assessed by an immuno-chromatography test (ICT) in people recruited from all five districts of Conakry. Mosquitoes were collected over a 1-year period, in 195 households in 15 communities. A proportion of mosquitoes were analysed for *W. bancrofti*, using dissection, loop-mediated isothermal amplification (LAMP) assay and conventional polymerase chain reaction (PCR).

**Results:**

CFA test revealed no infection in the 611 individuals examined. A total of 14,334 mosquitoes were collected; 14,135 *Culex* (98.6 %), 161 *Anopheles* (1.1 %) and a few other species. Out of 1,312 *Culex* spp. (9.3 %) and 51 *An. gambiae* (31.7 %) dissected, none was infected with any stage of the *W. bancrofti* parasite. However, the LAMP assay revealed that 1.8 % of *An. gambiae* and 0.31 % of *Culex* spp. were positive, while PCR determined respective prevalences of 0 % and 0.19 %.

**Conclusions:**

This study revealed the presence of *W. bancrofti* DNA in mosquitoes, despite the apparent absence of infection in the human population. Although MDA interventions are not recommended where the prevalence of ICT is below 1 %, the entomological results are suggestive of the circulation of the parasite in the population of Conakry. Therefore, rigorous surveillance is still warranted so that LF transmission in Conakry would be identified rapidly and adequate responses being implemented.

## Background

Annual mass drug administration (MDA) with single-dose diethylcarbamazine (DEC) or ivermectin (IVM), in combination with albendazole (ALB), for 4–6 years is the global strategy for the elimination of lymphatic filariasis [1]. While it may be perceived that achieving a proper MDA coverage might be easier in urban settings in Africa where the public health system usually is stronger (in comparison to rural areas), the implementation of MDA interventions can be a challenge under certain conditions in urban settings that are characterised by overcrowded slums, intense population movements, poor definition of risk factors, low levels of transmission, uncertainty to determine local transmission, etc. [2], particularly in view of the need to achieve at least 65 % effective coverage [3].

For many years, the diagnosis of bancroftian filariasis depended exclusively on the identification of microfilariae (MF) in blood specimens taken at night [4]. Likewise, studies on the prevalence of *Wuchereria bancrofti* in mosquitoes have traditionally relied on manual dissection and examination [5]. Other diagnostic tests have recently been developed that include sero-diagnostic methods based on the detection of circulating filarial antigens (CFA) [6], the detection of IgG4 antibody as a marker for patent MF and the polymerase chain reaction (PCR)-based detection of *W. bancrofti* DNA in mosquitoes and human blood sample [7]. These advances have allowed sampling during the day for humans and high-throughput analysis for both human and vector samples, and in turn increased the sensitivity of LF diagnosis.

The neglected tropical diseases (NTDs) master plan for Guinea reported the prevalence of CFA of *W. bancrofti* to range between 4.5 % and 46.3 % and that of MF between 3.0 % and 16.7 % [8]. However, rural–urban migration is an important risk factor of LF transmission in endemic countries [9]. Population movements may help spread LF infection from endemic to non-endemic areas where potential LF vectors are present, or may also lead to the resurgence of infection in areas under control [10]. Interpretation of the importance of infection rates in humans is also confounded by large movements of infected individuals from endemic to non-endemic areas, especially in conflict areas in West Africa, where some transient populations or internally displaced persons or refugees from the neighbouring countries settle in large cities not directly affected by conflict [11]. Conakry, the capital of Guinea, accounts for about one quarter of the total population of the country of whom most originated from other geographical regions of the country and neighbouring Côte d’Ivoire, Liberia and Sierra Leone [11, 12].

Establishing and maintaining transmission of LF in new areas depends on the presence of appropriate vectors and their capability to sustain transmission [10]. Thus, monitoring the LF infection in populations through mosquitoes (xenomonitoring) provides an important way of demonstrating potential transmission in an area, and has been suggested as a tool for monitoring the impact of MDA on LF transmission [13, 14]. To date, while good epidemiological data on LF exist in rural areas in Guinea, there have been no reports on the status of the infection in Conakry. A mapping study carried out in Kassa, an island close to Conakry in 2005 [8], indicated no infection. However, this finding might not be representative for the city of Conakry, due to demographic, ecologic and socioeconomic differences. In view of the paucity of epidemiological data, the World Health Organization (WHO) recommended a re-mapping of LF in Conakry. Hence, the study presented here was designed to obtain baseline data on the prevalence of *W. bancrofti* infection, coupled with entomological surveys, in order to determine whether or not LF transmission occurs in Conakry, which would require MDA.

## Methods

### Ethics statement

The surveys were conducted in accordance with the study protocol approved by the Institutional Ethics Review Board of the Liverpool School of Tropical Medicine (1189RS) and from the National Ethics Committee for Research in Health (CNERS) from the Republic of Guinea (20/CNERS/12). Written informed consent was obtained from individuals aged 18 years and above. For minors (aged <18 years), written informed consent was obtained from parents or legal guardians, while minors provided oral assent. Due to high illiteracy rate, in some households, oral rather than written informed consent was obtained. CNERS explicitly approved our consent procedures.

Participants were informed about the purpose and procedures of the study, including potential risks and benefits. The data were analysed and reported to exclude any directly identifiable information, in order to maintain anonymity of participants.

### Study sites

This study was carried out in Conakry, the capital of Guinea. The country is located at the coast in West Africa and shares borders with six countries: Côte d’Ivoire, Guinea Bissau, Liberia, Mali, Senegal and Sierra Leone [12]. Conakry is a peninsula of 308 km^2^, with a length of 34 km and a width of 1–6 km. According to a census done in 1999, the population was about 1.5 million, which is likely to have increased by more than 40 % taking into account annual growth rates. Conakry accounts for about a quarter of the total population of Guinea, and about 60 % of the urban population [15].

### Detection of *W. bancrofti* antigen in blood

**Fig. 1:**
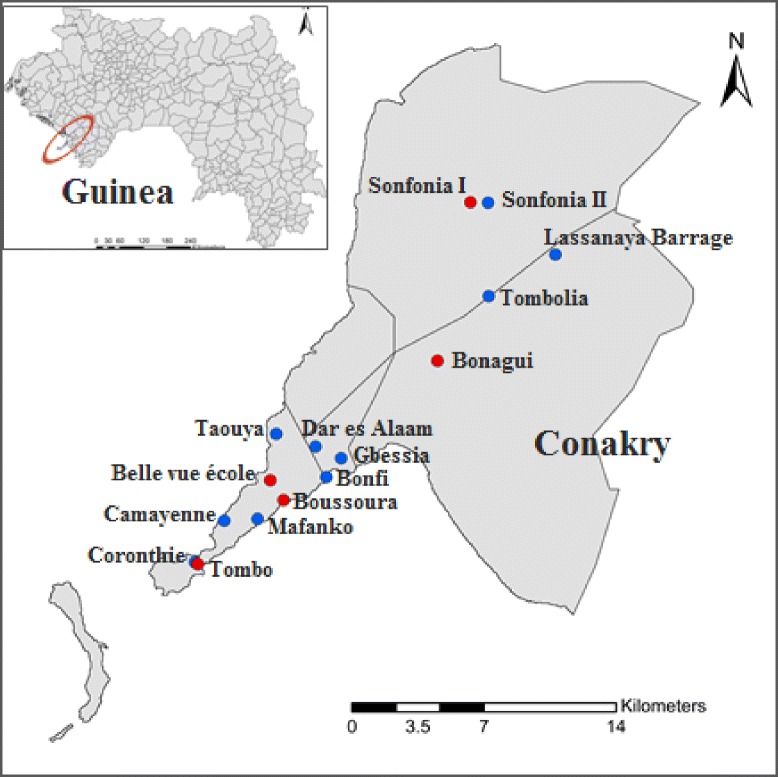
Survey locations for collection of mosquitoes in Conakry, Guinea, between December 2012 and November 2013. Blue circle, window exit trap and pyrethrum spraying catches; red circle, window exit trap, pyrethrum spraying catches and ICT test sites

A serological survey was carried out following the WHO mapping guidelines [16] with slight modifications, as follows. First, in order to cover the entire city, sample collection sites were chosen randomly in the five districts of Conakry (Fig. 1). Second, only individuals’ aged ≥15 years from the five districts were included in the survey. We employed an immunochromatography card test (ICT) (Alere, NOW, ICT filariasis kits; Binax, Portland, USA) for the detection of circulating filarial antigen in finger-prick blood samples taken during the day. All results were read after 10 min according to the manufacturer’s instructions. Clinical examination for signs of lymphoedema was conducted for all subjects.

### Mosquito collection

Using a map of the city, a survey of larval breeding sites was undertaken. Based on the information gathered, 15 sectors were chosen based on the potential exposure of the population to mosquito bites, according to information provided by district leaders and observation of risk factors associated to the presence of mosquito breeding sites. From this information, mosquito collection sites were selected to represent different sectors of the city (Fig. 1). This was to allow the collection of as many samples as possible. Mosquitoes were collected over a 1-year period from December 2012 to November 2013, using exit traps and pyrethrum knock-down spray sheet collections (PSC) [17] in different households. Collections were undertaken in 15 communities in all five districts of Conakry (Fig. 1). In each community, 9–10 days a month, exit traps were operated in 10 households. Exit traps and PSC were not performed in the same households. However, in case of unavailability, mosquitoes were collected in a neighbouring household. PSC collections were done once every month, between 06:00 and 09:00 h in three households.

### Mosquito processing and dissection

The collected mosquitoes were identified based on their morphological characteristics [18, 19]. All dissectible female specimens of the genera of *Anopheles* and *Culex* were analysed for parity, by dissecting and inspecting the ovaries and Malpighian tubes under a microscope [20] (Table 3). Ten to 15 % of mosquitoes collected from each site were also dissected for detection of *W. bancrofti* larvae. The head, thorax and abdomen were dissected separately on a glass slide in three drops of saline and examined under a compound microscope for larval detection [21]. The dissected mosquitoes were scraped into individual Eppendorf tubes and all other mosquitoes were also stored individually on silica gel and sent to the Noguchi Memorial Institute for Medical Research, University of Ghana (Accra, Ghana), for subsequent molecular analyses.

### Detection of *W. bancrofti* DNA in mosquitoes

*Culex* mosquitoes were grouped into pools, with a maximum of 20 mosquitoes per pool. *Anopheles* mosquitoes were analysed individually. DNA was extracted from the mosquitoes using the Qiagen DNeasy tissue kit (QiagenDNeasy^®^ kit; Mississauga, Canada). Identification of parasite DNA in the mosquitoes was done using the loop-mediated isothermal amplification (LAMP) assay [22] and a PCR method [23] for detection of *W. bancrofti*. The LAMP assay enables the formation of a turbid solution, indicative of product amplification, and sample confirmation can therefore be done visually, or through florescent detection under UV light directly in the Eppendorf tubes. The assays were performed as described by de Souza and colleagues [22]. All LAMP-positive samples were confirmed twice and PCR analysis was conducted to confirm samples positive for the LAMP assay. A negative and positive control was included in all LAMP and PCR assays.

### Statistical analysis

Data were entered onto a Microsoft Excel spreadsheet and transferred to STATA version 11 (Stata Corporation; College Station, United States of America). Pool screen version 2.0.3 [24] was used to calculate the maximum likelihood estimate of infection in the vector populations together with the associated 95 % confidence interval (CI). ArcGIS version 10.2.1 software was used for mapping the study site.

## Results

### Detection of *W. bancrofti* prevalence

A total of 611 individuals (347 females, 56.8 %) were sampled for the detection of CFA. None (0 %) of the subjects had detectable levels of circulating parasite antigen (Table 1). However, three cases of elephantiasis of the legs were identified (two males, one female; all aged >20 years), accounting for 0.5 % of all individuals sampled.

**Table 1:** Prevalence of *W. bancrofti* infection in Conakry, Guinea between December 2012 and November 2013, as estimated by ICT

District	No. sampled	Female	Male	No. of lymphoedema	ICT-positive
Matoto	153	91	62	1	0
Matam	104	48	56	2	0
Ratoma	114	79	39	0	0
Dixinn	122	74	48	0	0
Kaloum	118	55	59	0	0
Total	611	347	264	3 (0.5 %)	0 (0 %)

In this survey, microfilaremia was not determined since all individuals tested by ICT were negative.

### Mosquito collection and dissection

Overall, 14,334 mosquitoes were collected, of which 14,135 (98.6 %) were *Culex* and 161 (1.1 %) were *Anopheles* (Table 2). Other mosquito species, specifically *Mansonia uniformis* and *Aedes aegypti*, were also collected, but at very low numbers. Overall, 13,190 (93.3 %) of female *Culex* and 147 (91.3 %) of female *Anopheles* mosquitoes were analysed for parity, by dissecting and inspecting the ovaries and Malpighian tubes under a microscope. The parity rates in *Culex* and *Anopheles* mosquitoes were 46.5 % and 38.8 %, respectively (Table 3). Results of the dissections revealed no *W. bancrofti* larval stages in the head, thorax or abdomen of the examined *An. gambiae* and *Culex* mosquitoes (Tables 4 and 5).

**Table 2:** Species composition of mosquitoes collected using window exit traps and pyrethrum spray catches (PSC)

Genus	Species	Window exit traps	PSC	Total (%)
*Culex*	*decens*	5,751	4,868	10,619 (74.1)
	*quinquefasciatus*	1,668	1,812	3,480 (24.3)
	*annulioris*	2	2	4 (0.0)
	*poicilipes*	3	3	6 (0.0)
	*nebulosus*	1	2	3 (0.0)
	*ingrami*	17	4	21 (0.15)
	*cinereus*	0	2	2 (0.0)
*Culex sub-total*				14,135 (98.6)
*Anopheles*	*gambiae*	121	40	161 (1.1)
*Aedes*	*aegypti*	20	7	27 (0.19)
*Mansonia*	*uniformis*	11	0	11 (0.08)
Total		7,594	6,740	14,334 (100)

**Table 3:** Distribution of mosquitoes collected from Conakry, Guinea between December 2012 and November 2013

		*Culex*				*Anopheles*			
District	Community	No. of mosquitoes collected	Dissected for parity	No. nulliparous	No. parous	No. of mosquitoes collected	Dissected for parity	No. nulliparous	No. parous
Matoto	Tombolia	1,261	1,079	622	457	0	0	0	0
	Gbessia	1,320	1,261	533	728	0	0	0	0
	Bonagui	665	617	280	337	0	0	0	0
	Lassanaya	535	499	269	230	8	8	1	7
Matam	Boussoura	1,247	1,193	601	592	4	3	2	1
	Bonfi	731	710	386	324	0	0	0	0
	Mafanko	600	564	289	275	0	0	0	0
Ratoma	Taouya	1,085	1,036	660	376	4	4	3	1
	Dar es Alaam	915	811	395	416	1	1	0	1
	Sonfonia I	633	609	361	248	107	100	62	38
	Sonfonia II	710	673	459	214	28	23	18	5
Dixinn	Belle Vue	1,954	1,812	990	822	3	3	3	0
	Camayenne	701	681	393	288	0	0	0	0
Kaloum	Tombolia	1,497	1,397	712	685	2	2	1	1
	Coronthie	281	248	104	144	4	3	0	3
	Total	14,135	13,190 (93.3 %)	7,054 (53.5 %)	6,136 (46.5 %)	161	147 (91.3 %)	90 (46.5 %)	57 (38.8 %)

**Table 4:** *Wuchereria bancrofti* infection rates in *An. gambiae s.l.* collected from Conakry, Guinea between December 2012 and November 2013

		Dissection			Molecular methods		
District	Community	No. collected	No. dissected	No. positive (%)	No. examined	LF LAMP-positive (%)	PCR-positive (%)
Matoto	Tombolia	0	-	-	0	-	-
	Gbessia	0	-	-	0	-	-
	Bonagui	0	-	-	0	-	-
	Lassanaya	8	4	0 (0)	4	(0)	0 (0)
Matam	Boussoura	4	2	0 (0)	2	0 (0)	-
	Bonfi	0	-	-	0	-	-
	Mafanko	0	-	-	0	-	-
Ratoma	Taouya	4	3	0 (0)	1	0 (0)	-
	Dar es Salaam	1	0		1	0 (0)	-
	Sonfonia I	107	26	0 (0)	81	1 (1.2)	0 (0)
	Sonfonia II	28	10	0 (0)	18	1 (5.6)	0 (0)
Dixinn	Belle Vue	3	3	0 (0)	0	-	-
	Camayenne	0	-	-	0	-	-
Kaloum	Tombo	2	2	0 (0)	2	0 (0)	-
	Coronthie	4	1	0 (0)	3	0 (0)	-
Total		161	51	0 (0)	112	2 (1.8)	0 (0)

**Table 5:** *W. bancrofti* infection rates in *Culex* species collected from Conakry, Guinea between December 2012 and November 2013

		Dissection			Pool screening					
District	Community	No. collected	No. dissected	Positive (%)	No. examined	Pools examined	LAMP-positive (MLE %)	95 % CI	PCR-positive (MLE %)	95 % CI
Matoto	Tombolia	1,261	79	0	240	12	0 (0)	-	-	-
	Gbessia	1,320	149	0	240	12	0 (0)	-	-	-
	Bonagui	665	88	0	240	12	0 (0)	-	-	-
	Lassanaya	535	30	0	287	15	1 (0.96)	(0.11–3.36)	1 (0.96)	(0.11–3.36)
Matam	Boussoura	1,247	110	0	240	12	0 (0)	-	-	-
	Bonfi	728	53	0	240	12	3 (1.43)	(0.27–4.11)	2 (0.91)	(0.11–3.16)
	Mafanko	600	55	0	271	14	1 (0.38)	(0.01–1.95)	1 (0.38)	(0.01–1.95)
Ratoma	Taouya	1,085	57	0	260	13	0 (0)	-	-	-
	Dar es Alaam	915	118	0	240	12	0 (0)	-	-	-
	Sonfonia I	635	48	0	237	12	0 (0)	-	-	-
	Sonfonia II	711	61	0	191	10	0 (0)	-	-	-
Dixinn	Belle Vue	1,954	164	0	240	12	2 (0.91)	(0.11–3.16)	2 (0.55)	(0.11–3.16)
	Camayenne	701	43	0	278	14	1 (0.37)	(0.01–1.90)	1 (0.37)	(0.01–1.90)
Kaloum	Tombo	1,497	212	0	240	12	0 (0)	-	-	-
	Coronthie	281	45	0	191	10	3 (1.86)	(0.36–5.38)	0 (0)	–
Total		14,135	1,312	0 (0 %)	3,635	184	11 (0.31)	(0.15–0.57)	7 (0.19)	(0.07–0.41)

### Detection of *W. bancrofti* DNA in mosquitoes

Overall, 112 *Anopheles* mosquitoes were analysed individually, and 3,635 *Culex* were sorted into 184 pools with a range of 7–20 mosquitoes per pool. Of the mosquitoes processed, 2 (1.8 %) *An. gambiae* and 11 pools of *Culex* were found positive (Tables 4 and 5), when subjected to a LAMP assay. The pool screening calculation indicated a maximum likelihood estimate (MLE) of infection of 0.31 % (95 % CI: 0.15–0.57 %) for *Culex*. All LAMP-positive samples were further analysed using PCR. The two LAMP-positive *An. gambiae* samples were negative by PCR. Seven of the 11 *Culex* pools positive by LAMP were also positive by PCR. The pool screening calculation indicated a MLE of 0.20 % (95 % CI: 0.07–0.41 %).

## Discussion

A cross-sectional ICT antigen detection survey carried out in all five districts of the capital city of Guinea revealed that none of the 611 individuals aged ≥15 years had detectable levels of CFA. This is in accordance with the result of a study performed in 2005 at Kassa, an island community of Conakry [8], which also reported a zero prevalence using the same diagnostic approach. Based on these results and WHO recommendations to treat only those endemic areas where the prevalence is above 1 %, the city of Conakry does not qualify for MDA.

To demonstrate the potential for active transmission in this urban environment, *An. gambiae* and *Culex* mosquitoes were collected and processed for detection of *W. bancrofti* infection, using standard dissections, followed by more sophisticated molecular methods. Importantly, none of the dissected mosquitoes was found positive. However, some mosquitoes further processed by molecular tests were found positive. Arguments have been made against dissection as opposed to molecular methods for monitoring transmission. A major constraint of dissections is the difficulty to detect MF and third-stage larvae (L_3_) [14, 25] and the large numbers of mosquitoes that need to be processed to find infections in areas with low endemicity [14, 26]. Thus, there has been an increased interest in molecular-based detection methods that can either use individual mosquitoes or pools, resulting in rapid, high-throughput screening of mosquito vectors [26, 27]. Our study confirms that molecular methods are more sensitive than standard dissection for the detection of infections in mosquito vectors [28].

Very low numbers of *An. gambiae* were collected in this study. While this might bring into question the effectiveness of the collection method, it is important to note that 12 monthly collections were undertaken and the same methods were used for the collection of both *Anopheles* and *Culex* mosquitoes. Mosquitoes were collected by two different methods, namely window exit traps and PSC [17]. The latter has been widely used in sampling *Anopheles* mosquitoes in malaria intervention programmes [29] and remains the ‘gold’ standard for collecting indoor-resting, blood-fed and gravid mosquitoes [29, 30]. In West African cities and urban areas with high pollution levels, *Culex* mosquitoes are the predominant mosquito species [22, 31]. Likewise, the use of the same collection methods in rural areas reveals higher proportions of *Anopheles* compared to *Culex* [32]. As such, the low numbers of *An. gambiae* collected in this study cannot be attributed to the collection method, but rather to the vector ecology. A survey of the breeding sites in Conakry revealed these to be heavily polluted and presenting ideal conditions for the breeding of *Culex*, compared to *Anopheles*, which prefer clean breeding sources.

The results from the LAMP and PCR assays may suggest that the LAMP has higher sensitivity as an epidemiological tool for detecting *W. bancrofti* infection compared to PCR, although previous reports have shown them to be substantially similar [33]. The LAMP uses four primers (six distinct sequences) that are simultaneously used to initiate DNA synthesis from the original unamplified DNA to generate a stem-loop DNA for subsequent LAMP cycling. Thus, target selectivity is higher than in conventional PCR [34, 35]. In addition to high sensitivity, the LAMP is also less prone to the presence of irrelevant DNA and inhibition compared to PCR and has the advantage of shorter reaction time, simple readout system and the use of cheaper technology tools [34–36]. As such, it enjoys increasing popularity for the diagnosis of a host of diseases [22, 37, 38]. Indeed, LAMP represents a powerful tool that can be employed in the evaluation of LF control activities, especially in the end-game when interruption of LF transmission must be certified. However, the epidemiological usefulness of the LAMP assay must be determined in carefully thought out studies, with sufficiently high numbers of vector mosquitoes.

Various studies conducted in different localities in West Africa have identified *An. gambiae* as the main LF vector species [39–41]. However, in our collections, the proportion of *An. gambiae* among all the mosquitoes caught was low, and hence, the infection rate must be interpreted with caution. Indeed, very high numbers of mosquitoes should be analysed in settings characterised by low levels of infection in the human population, so that very low levels of infection in the vectors can be determined [42]. Moreover, for parasitic diseases and more specifically LF whose infection is related to the parasite load and thus the frequency of infective bites [43], infection in an urban system characterised by a low vector density may not lead to the transmission of *W. bancrofti*. Nonetheless, given that LAMP identified two *An. gambiae* mosquitoes in Conakry as positive, is it not possible to rule out transmission without further detailed entomological and parasitological studies, which calls for continuous, rigorous surveillance.

While *Culex* mosquitoes might not play a role in LF transmission in West Africa [22, 40, 44], our results suggest that *Culex* mosquitoes are capable of ingesting parasite material while feeding on MF-positive individuals, demonstrating the potential of using non-vector species as a proxy for determining the presence of LF in human populations. Fischer and colleagues [45] showed in laboratory experiments that parasite DNA can be detected in both vector and non-vector mosquitoes for 2 weeks or longer after they ingest MF-positive blood. Thus, the detection of *W. bancrofti* DNA in mosquitoes confirms that infected individuals are present in Conakry. This indicates that transmission from mosquitoes to humans may occur, albeit at very low levels. Outbreaks of *Culex* mosquitoes in urban cities in West Africa have coincided with the development of large cities, which has taken an extraordinary expansion in recent decades [9, 46, 47]. Interestingly, in 1981, Fain reported that in West African regions, local strains of *W. bancrofti* have not yet fully adapted to *Culex* mosquitoes to facilitate transmission [48], but this claim requires further scientific inquiry.

We were unable to demonstrate ongoing transmission of LF based on infection rates in humans and mosquitoes. Nevertheless, the presence of two infected *An. gambiae* vectors using a diagnostic method that is not stage-specific implies that people may be at risk of infection [23]. However, the prevalence of parasite DNA in mosquitoes does not necessarily imply that LF transmission is ongoing in a given setting [14]. Studies by Farid and colleagues showed that mosquitoes that fed on people with very low levels of MF sometimes ingested MF but rarely produced infective larvae [49]. However, to ascertain the transmission of LF in areas where positive mosquitoes were detected, we recommend the use of gravid traps for the collection of mosquitoes [50], followed by stage-specific molecular detection methods for *W. bancrofti* [51]. This study further points to the focal nature of LF transmission, and the usefulness of xenomonitoring. As shown in the present investigation, analysing 611 individuals out of a population of over 1.5 million inhabitants (based on logistical, programmatic and ethical considerations) may be grossly inadequate to determine the presence of infection in areas with low infection levels in the population. As such, the widely used methodology in assessing the transmission of LF in areas with low infection rates requires critical review.

## Conclusions

Our study demonstrated the presence of *W. bancrofti* DNA in mosquitoes in Conakry, despite the apparent absence of circulating filarial antigen in the human population. The study also demonstrated the utility of the LAMP assay in xenomonitoring. Based on the findings reported here and on WHO recommendations to undertake MDA in implementation units with prevalence of 1 % or more, the city of Conakry does not qualify for MDA. However, rigorous surveillance through testing of a much larger human population sample size and entomological surveys, is required to monitor the epidemiological situation of LF in Conakry as well as inform future decisions.
